# Natural and human‐induced environmental changes and their effects on adaptive potential of wild animal populations

**DOI:** 10.1111/eva.12928

**Published:** 2020-02-14

**Authors:** Dany Garant

**Affiliations:** ^1^ Département de biologie Faculté des Sciences Université de Sherbrooke Sherbrooke QC Canada

**Keywords:** birds, environmental heterogeneity, evolution, genetic variance, mammals, plasticity, selection

## Abstract

A major challenge of evolutionary ecology over the next decades is to understand and predict the consequences of the current rapid and important environmental changes on wild populations. Extinction risk of species is linked to populations’ evolutionary potential and to their ability to express adaptive phenotypic plasticity. There is thus a vital need to quantify how selective pressures, quantitative genetics parameters, and phenotypic plasticity, for multiple traits in wild animal populations, may vary with changes in the environment. Here I review our previous research that integrated ecological and evolutionary theories with molecular ecology, quantitative genetics, and long‐term monitoring of individually marked wild animals. Our results showed that assessing evolutionary and plastic changes over time and space, using multi‐trait approaches, under a realistic range of environmental conditions are crucial steps toward improving our understanding of the evolution and adaptation of natural populations. Our current and future work focusses on assessing the limits of adaptive potential by determining the factors constraining the evolvability of plasticity, those generating covariation among genetic variance and selection, as well as indirect genetic effects, which can affect population's capacity to adjust to environmental changes. In doing so, we aim to provide an improved assessment of the spatial and temporal scale of evolutionary processes in wild animal populations.

## 1 INTRODUCTION

Environmental changes are currently occurring at an unprecedented rate, mainly due to human activities (Fey et al., 2015; IPCC, 2014). One of the greatest challenges faced by biologists presently and over the next decades is to understand and predict the consequences of these changes on biodiversity (Pacifici et al., 2015; Parmesan, 2006). The impacts of such changes on evolutionary processes, which produce and maintain biodiversity, however, still need to be further evaluated. In this context, there is an urgent need to provide accurate assessment concerning evolutionary potentials of species in changing environments (Hoffmann & Sgro, 2011; Smith, Kinnison, Strauss, Fuller, & Carroll, 2014).

Two main evolutionary‐related processes will allow a population to face and adapt to its changing environment. First, phenotypic plasticity of traits (e.g., the ability of an individual genotype to produce different phenotypes across different environments; Stearns, 1989) is a process that should typically act rapidly and on shorter terms to allow individuals to adjust their phenotypes to reproduce and survive in new conditions (Chevin, Lande, & Mace, 2010). Secondly, an evolutionary change will be required on the mid‐ and long‐term if a population is to adapt to new conditions. However, for this to occur, additive genetic variance must be sufficient (Bürger & Lynch, 1995), as it will allow selection to bring the population to its new optimum. Studies of species adaptive capacity are critical since extinction risk is tightly linked to a species’ evolutionary potential and to its ability to express adaptive phenotypic plasticity: Species with low adaptive potential or with reduced plasticity have lower chance of persistence in a changing environment (Lande & Shannon, 1996; Merilä & Hendry, 2014).

Previous studies assessing evolutionary change in face of changing environments over time or space have allowed significant advances in our current knowledge, but have also reported equivocal results (reviewed in Merilä, 2012; see also Merilä & Hendry, 2014). This is partly because predicting evolution across different temporal and spatial scales is particularly challenging (Reed, Schindler, & Waples, 2011; Robinson et al., 2009) and requires detailed long‐term datasets collected over broad geographic areas (Clutton‐Brock & Sheldon, 2010). For example, a few studies using such approach greatly helped decipher if changes observed are genetic or plastic in nature. For instance, Teplitsky, Mills, Alho, Yarrall, and Merilä (2008) showed that the change in body size observed in red‐billed gulls (*Larus novaehollandiae scopulinus*) over time, which was previously interpreted as evidence for genetic adaptation to a warming climate in the framework of Bergmann's rule, was instead mainly the result of phenotypic plasticity.

Also, several studies of evolutionary changes have focussed on single‐trait analyses (see Blows & Hoffmann, 2005 for details). Univariate approaches are unrealistic given the inherently multivariate nature of selection and phenotypic variation and because of the possible presence of genetic correlations among traits that could constrain their evolution (Agrawal & Stinchcombe, 2009; Chevin, 2013). Using proper environmental parameters to assess their effect on evolutionary processes is also critical. A recent study conducted by Hayward et al. (2018) in Soay sheep (*Ovis aries*), using six morphological and life‐history traits, showed that selection pressures acting on these trait may be importantly affected by environmental conditions (i.e., presence of Selection by Environment interaction: S X E). On the other hand, Hayward et al. (2018) found little evidence that environmental variation affected genetic variance for the same traits (no Genetic by Environment interaction: no G X E). However, similarly to most studies they reviewed (see table 1 in Hayward et al., 2018), Hayward et al. (2018) used a single environmental variable (population density) to perform their analyses. Interestingly, other recent examples also showed opposite effect of environment on evolutionary parameters. For example, genetic variance for fitness varied among environmental contexts (i.e., presence of G X E) in the partridge pea *Chamaecrista fasciculata* (see Sheth, Kulbaba, Pain, & Shaw, 2018). Also, a recent meta‐analysis (see Siepielski et al., 2019) found no evidence that warmer environments were associated with selection for smaller size across several taxonomic groups (i.e., no S X E).

To improve our understanding of the determinants of plasticity and evolutionary potential across traits and populations, it is thus vital: (a) to quantify strength, direction, and variation in selective pressures, quantitative genetics parameters, and phenotypic plasticity, (b) to do so for multiple traits at the same time in wild populations, and (c) to achieve this across several years, under different environmental conditions and also using different environmental variables. These were the central goals of my research program over the last 15 years (see also Box 1). To do so, my research team and I integrated ecological and evolutionary theories with state‐of‐the‐art statistical techniques, molecular ecology, quantitative genetics, and monitoring of individually marked wild animals. Over the next section, I provide a brief overview of the specifics of our approach.

Box 1 **Louis Bernatchez: a great mentor, an exceptional scientist, and an even better friend!**

First and foremost, I need to say that I have been really fortunate to be part of Louis’ laboratory and even more to benefit from his mentoring and enjoy his support and friendship over the last 25 years (time flies)! I had the privilege to complete both my MSc and PhD degrees under the supervision of Louis, co‐supervised by Julian Dodson, at Université Laval. Right from the start, I knew I was in the best place possible to expand my way of thinking about science and improve and expand my research skills. Based on my supervisors original ideas, my MSc research focussed on within‐river population structure in Atlantic salmon (*Salmo salar*) (not a sturgeon!), testing the theoretical framework of the member/vagrant hypothesis proposed by Sinclair (1988). Our results showed that salmons not only distribute themselves among rivers in a structured fashion, but that small‐scale within‐river population structure may also be prevalent. This work was not well received at first by some journals and editors (!), but Louis, through his unique supervising style, encouraged me to persevere and we finally published this work (Garant, Dodson, & Bernatchez, 2000), which has now been cited more than 200 times (Google scholar, January 2020). Then followed my PhD research, four great years of fun and science, also conducted on Atlantic salmon and focussing on the determinants of reproductive success in this species. Due to a great experimental set‐up and collaborations with people from the *Centre Interuniversitaire de Recherche sur le Saumon Atlantique* (CIRSA), we provided one of the first genetic evaluation of the species’ mating system in the wild (Garant, Dodson, & Bernatchez, 2001). We also reported variable growth and survival of male alternative mating tactics (Garant, Fontaine, Good, Dodson, & Bernatchez, 2002), and of differential reproductive success and genetic basis of these mating tactics (Garant, Dodson, & Bernatchez, 2003; Garant, Fleming, Einum, & Bernatchez, 2003). All this work was very innovative and has been cited more than 470 times (Google scholar, January 2020). Louis's laboratory was also a great place to establish collaborations. For example as a side project of my PhD, I collaborated with Christian Landry (who is now a Professor at U. Laval and holds a Canada Research Chair in Evolutionary Cell and Systems Biology) to show that mate choice was influenced by MHC genes in salmon (Landry, Garant, Duchesne, & Bernatchez, 2001). Luckily for me, my collaboration with Louis was maintained after my graduate studies and I benefited from his excellent advices during my first years as a Professor at Université de Sherbrooke. Among other things, we organized a symposium sponsored by *Evolutionary Applications* in Torino in 2009, as part of the European Society for Evolutionary Biology (ESEB) meeting. We have also been involved in several research projects together over the last years, co‐supervising graduate students and producing several contributions related to the impact of stocking in wild populations of Brook trout (*Salvelinus fontinalis*) (see Marie, Bernatchez, & Garant, 2010, Lamaze, Sauvage, Marie, Garant, & Bernatchez, 2012, Létourneau et al., 2018 and Gossieaux, Bernatchez, Sirois, & Garant, 2019 e.g.,). We are still collaborating on a research project aiming to assess the impact of climate change on Brook trout and I look forward to more collaborations and continued friendship in the future. Merci mon ami!!

## 2 LONG‐TERM STUDY SYSTEMS AND GENERAL METHODS

Relatively few studies of wild vertebrates have obtained the long‐term detailed data required to explain the evolutionary dynamics of quantitative traits in the wild (reviewed in Clutton‐Brock & Sheldon, 2010). To achieve our research objectives, my research team and I used exceptional long‐term datasets including several hundred marked individuals, with information on phenotypes and relatedness obtained over many generations and in contrasting environmental conditions for Tree swallow (*Tachycineta bicolor*) and Eastern chipmunk (*Tamias striatus*).

### 2.1 Long‐term study systems

#### 2.1.1 Tree swallows

Since 2004, we monitor a population of Tree swallow within a study system comprising 400 nest boxes distributed among 40 farms, over a 10,200 km^2^ area in southern Québec (see Ghilain & Bélisle, 2008 for details). Every year, all breeding attempts in nest boxes are surveyed and measures of all key traits are taken, from egg‐laying until all nestlings have fledged. Each bird is individually ringed: Chicks are marked at 12 days after hatching, while adults are captured and marked at nest boxes either when incubating (females) or feeding young (males). Blood samples are collected from all individuals for molecular ecology analyses (see below; see also details in Bourret & Garant, 2017; Lessard, Bourret, Bélisle, Pelletier, & Garant, 2014). On each farm, we also collect flying insects using combined window/water‐pan flight traps (see Bellavance, Bélisle, Savage, Pelletier, & Garant, 2018 for details). This system offers excellent opportunities to investigate the effects of environmental heterogeneity on a large sample size because nest boxes are located in habitats ranging from extensive agriculture lands (high‐quality habitats, where more fledglings are produced) to intensive agricultural cultures (low‐quality habitats, fewer fledglings produced).

#### 2.1.2 Eastern chipmunks

This study system consists of 4 fixed trapping grids located in southern Québec, where a population of eastern chipmunk is monitored since 2005 (1 grid from 2005–2010 and 3 grids from 2011‐present). The grids are located in a deciduous forest where we trap chipmunks every week from mid‐May to mid‐September. At each capture, every animal is aged, weighed, sexed, and uniquely marked with ear tags. Behaviors are measured for each individual using standard open field (Montiglio, Garant, Thomas, & Réale, 2010) and handling bag (Montiglio, Garant, Pelletier, & Réale, 2012) tests. Ear tissues are collected from all marked individuals for molecular ecology analyses (see below; see details in Bergeron, Réale, Humphries, & Garant, 2011b; Chambers & Garant, 2010). Autumn masts of American beech (*F. grandifolia*), the dominant canopy tree on our sites and the main source of storable food items for chipmunks, are quantified using seed collectors distributed on the trapping grids (Bergeron, Réale, Humphries, & Garant, 2011a). Beech seed production displays extreme annual fluctuations linked to pulsed resources, which results in important differences in habitat quality between years (Bergeron et al., 2011a). This system thus also offers great opportunities to assess the effects of environmental variability on a wild population.

### 2.2 General approach

#### 2.2.1 Quantification of environmental conditions

In each study system, the quantification of the relevant ecological variables linked to habitat variability, and thus environmental changes, is performed over space and time. Weather and population‐specific variables typically affecting reproductive success and survival of our studied species are monitored on a yearly basis: daily temperature and rainfall, ectoparasite levels for each brood/litter, and local breeding density. Importantly, food availability on each study sites is quantified through monitoring of insects abundance (e.g., mainly dipterans) for tree swallows (Bellavance et al., 2018) and American beech seeds for chipmunks (Bergeron et al., 2011a). For tree swallows, an annual quantification of the level of agricultural exploitation is conducted using GIS software to measure the proportion of each type of agriculture at different spatial scales (e.g., 500 m, 5 km) around each nest box. For chipmunks, a detailed characterization of microhabitat vegetation around burrows of each individual residing on the grid is also available for several years.

#### 2.2.2 Molecular ecology and quantitative genetics analyses

Molecular ecology techniques are central to our research. In both study systems, extra‐pair paternity is very frequent: Mixed paternity occurs in 25 to 100% of litters in chipmunks (Bergeron et al., 2011b) and >80% of broods contain at least one extra‐pair offspring in tree swallows (Lessard et al., 2014). Therefore, using DNA microsatellite loci (see Chambers & Garant, 2010; Porlier, Bélisle, & Garant, 2009 for details) is essential to confirm maternity, assign paternity, identify siblings within each brood/litter, and obtain accurate estimates of the reproductive success (see Lessard et al., 2014) and mating patterns of adults (see Garant et al., 2018).

Microsatellite analyses also allow building multigenerational pedigrees using maternal, paternal, and sibling identities (Garant & Kruuk, 2005). Detecting and explaining the presence or absence of evolutionary responses to selection requires analytical techniques that can effectively partition the components of phenotypic variance in complex datasets. The now well‐established “animal model” was successfully applied to assess the evolutionary dynamics of traits in different populations (Kruuk, Charmantier, & Garant, 2014). Animal model analyses use the relatedness between all pairs of individuals in a multigenerational pedigree and allow estimating the variance components of traits, as well as covariances between traits, even for highly unbalanced datasets, thereby displaying whole genetic matrices (Kruuk et al., 2014).

## 3 UNDERSTANDING EVOLUTION IN ITS ECOLOGICAL CONTEXT

Over the next sections, I summarize achievements regarding three main interconnected research axes of my research program that provided advances in the understanding of evolution in its ecological context. First, we assessed the genetic architecture of important ecological traits and their variability depending on effect of context and environmental conditions. Second, we determined the environmental drivers underlying the variability of selection over temporal and spatial scales for both adults and juveniles. Third, we quantified the effect of variability of phenotypic plasticity of key traits on adaptation to changing environments. To illustrate this, I provide recent examples, mainly from our work on tree swallows and eastern chipmunks, achieved over the last few years. However, since I have also been privileged to collaborate with students and researchers using long‐term datasets of Blue tit (*Cyanistes caeruleus*) in France, I will complement this summary of our contributions using examples from this study system as well.

### 3.1 Genetic architecture of traits, context, and environmental conditions

Constraints on evolution of traits may arise due to low amount of genetic variance or to the presence of genetic correlations among traits (Morrissey, Walling, et al., 2012b; Teplitsky et al., 2014). Furthermore, the importance of these constraints may vary with developmental stage (see e.g., Parker & Garant, 2005) or environmental conditions (Sgrò & Hoffmann, 2004). For instance, harsher conditions are predicted to result in lower additive variance and greater environmental variance (reviewed in Charmantier & Garant, 2005). Problems may however also result from limited power of datasets to detect these quantitative genetic parameters (Pujol et al., 2018).

Several achievements were fulfilled within this research axis. First, we advanced our understanding of the applicability and reliability of quantitative genetics methods in the wild (see Bourret & Garant, 2017). To do so, we provided an assessment of the effects of identity errors, structure, and size of a pedigree on reliability of quantitative genetic estimates. In brief, we used empirical data from our tree swallow dataset to underline the bias—on heritability (*h* ^2^), coefficient of additive genetic variation (CV_A_), and genetic correlation (*r*_A_) estimates—resulting from using a “social” instead of a “genetic” pedigree in system with a high rate of extra‐pair paternity (EPP). Then, we also used simulations to show that power of datasets may be an issue for adult traits in populations with low recruitment (around 1% of nestlings), resulting in low connectivity in the pedigree, despite having several thousand individuals in the dataset. This, however, was not an issue for nestling traits in our tree swallow population, given that the high rate of EPP results in several half‐siblings and increased power.

**Figure 1:**
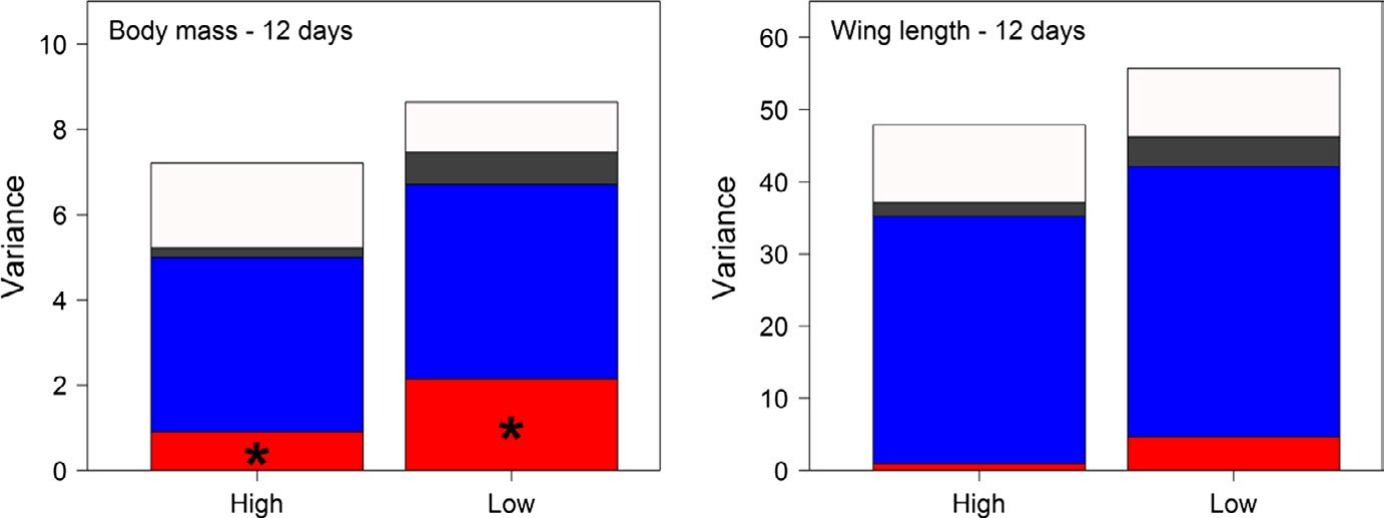
Variance components in low and high extensive landscapes (at 500 m around nest boxes) for body mass and wing length of nestlings in Tree swallows. Additive genetic (in red), brood (blue), and year (black) component of variance were obtained using a bivariate animal model. *p < .05. See Bourret et al. (2017) for methodological details

Secondly, we followed up on these previous results and assessed the evolutionary potential of morphological traits for juveniles of tree swallows across their nesting development (Bourret, Bélisle, Pelletier, & Garant, 2017). Our quantitative genetic analyses revealed fluctuations in selection acting on body mass and wing length of nestlings, as well as minor changes in heritability and additive genetic variation of these traits during their development. Together, these changes resulted in different predicted evolutionary responses depending on the life stage, but also depending on if we used the breeder equation or the secondary theorem of selection to predict the expected change in mean phenotype between two generations (see also Morrissey, Parker, et al., 2012a). Our results from that study were inconclusive on whether harsher conditions lowered additive variance while increasing environmental variance (as in Charmantier & Garant, 2005). However, additional recent analyses seem to suggest that, contrary to our expectations, while variance components seem to differ among types of habitats, poorer conditions (i.e., less extensive agricultural lands) may result in greater additive variance for morphological traits of nestlings in our study system (see Figure 1). This result remains to be further explored in future analyses.

Finally, we also conducted a study across contrasted habitat conditions and assessing spatial variation in G‐matrices for morphological and life‐history traits, using data from one continental and three island populations of Blue tit (Delahaie et al., 2017). These populations display marked phenotypic differences in both types of traits (see Charmantier, Doutrelant, Dubuc‐Messier, Fargevieille, & Szulkin, 2016) and are living in contrasted environments (i.e., deciduous or evergreen oak) with different food resources (see Blondel et al., 2006 for details). Interestingly, however, despite these important differences among populations at phenotypic and ecological levels, results of our study showed little support for a strong effect of environmental conditions on G‐matrices. Indeed, these were stable across populations for both type of traits, suggesting similar evolutionary potential in all populations.

### 3.2 Environmental drivers of the variability of selection over time and space

A critical parameter needed to assess the evolutionary potential of a population is the strength of selection acting on traits and combination of traits (Blows & Hoffmann, 2005; Teplitsky et al., 2014). In particular, patterns of selection may be affected by contrasted spatial and temporal environmental conditions that modify the expected evolutionary response (Siepielski et al., 2013; Tarwater & Beissinger, 2013). Comparisons of patterns across these scales provide accurate assessments of the environmental sensitivity of selection and a vital assessment of the extent of microgeographic adaptation and of its underlying driving mechanisms in the wild (Richardson, Urban, Bolnick, & Skelly, 2014).

Again, we completed several studies within this research axis. We first provided estimates of selection for key morphological and life‐history traits in tree swallow females (Millet, Pelletier, Bélisle, & Garant, 2015). We showed that fecundity selection until the hatchling stage fluctuated in strength/direction, over a period of ten years, for reproductive traits such as laying date and clutch size. However, this pattern was not observed during the nestling stage, which emphasized the need to consider how selection events may be fluctuating not only in time but also among different life‐history stages.

Then, in blue tits, we showed that climate change may greatly affect populations, as both temperature (Marrot, Charmantier, Blondel, & Garant, 2018) and extreme climatic events (Marrot, Garant, & Charmantier, 2017) are modulating the extent to which selection is acting within a population of this species. In details, we first showed that, like in tree swallows, selection acting on laying date fluctuated in magnitude and sign across a period 24 years in the so‐called Rouvière population (southern France). Importantly, we also showed that warmer spring temperatures were associated with stronger selection pressures for reproductive timing in this population, increasing the strength of selection by 46% for every +1°C anomaly (Marrot et al., 2018). In a study of the same population, we also explored the effects of extreme events, in terms of either heavy rainfall or high/low temperatures, on fitness and selection acting on clutch size and laying date (Marrot et al., 2017). We showed that fitness was negatively correlated with extremely hot days and that such events also increased the strength of selection acting on laying date: When 10% of broods in the population experienced this type of extreme weather, selection for earlier breeding increased by 39%.

Finally, we also performed analyses of selection acting on behavioral traits (docility and exploration) in eastern chipmunks and assessed the effects of fluctuating food resources and temporal variability on the patterns observed. Contrary to our predictions—we expected variability in selection through time linked to changes in food abundance associated with masting trees—we found no evidence of fluctuating selection on exploration (Bergeron et al., 2013) or docility (St‐Hilaire, Réale, & Garant, 2017). However, we found disruptive selection on adult exploration and docility behaviors, as individuals with either low or high exploration/docility scores had much higher survival probability than others (Bergeron et al., 2013; St‐Hilaire et al., 2017). Both studies thus highlight that disruptive selection may play an important role in the maintenance of phenotypic variance of behavioral traits in wild populations.

### 3.3 Variability and limits of phenotypic plasticity in different environments

Although important in the current context of environmental change, the effects of spatial and temporal habitat variability and the associated limits imposed on phenotypic plasticity in natural populations are still largely unknown (Boutin & Lane, 2014; Charmantier & Gienapp, 2014). This is mainly because relatively few studies had data available to perform detailed analyses at the individual level. To fill this gap, we used our long‐term datasets to assess the extent of variation in plasticity among (Porlier et al., 2012) and within (Bourret, Bélisle, Pelletier, & Garant, 2015) populations for breeding dates. In doing so, we specifically focussed on assessing if poorer environmental conditions may impose limits on the extent of phenotypic plasticity expressed by individuals (see review in Auld, Agrawal, & Relyea, 2010).

We first showed that plasticity in breeding date was variable among four populations of blue tits differing in habitat conditions. More specifically, we showed that while there was earlier onset of breeding in warmer years in all populations, there was a reduced extent of plasticity in less predictable environmental conditions (Porlier et al., 2012). We also found that significant inter‐individual variation in plasticity for laying date was only detected in populations where selection for earlier laying date was weaker (i.e., not statistically significant). This result potentially suggests an interaction between selection acting on a trait and plasticity patterns associated with this trait that may affect the short‐term adaptive potential of populations.

**Figure 2:**
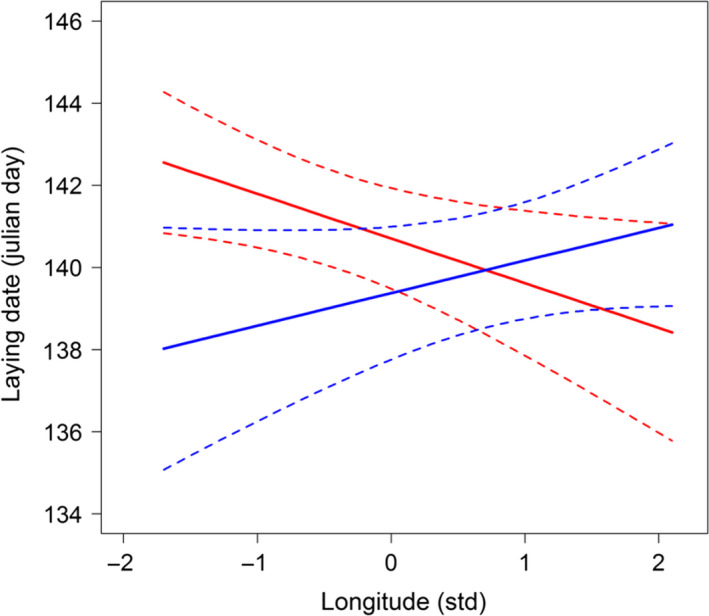
Plasticity as function of longitude in low (red) and high extensive (blue) agricultural landscapes (at 500 m around nest boxes) for laying dates of females in Tree swallows. See Bourret et al. (2015) for methodological details

In our tree swallow population, we showed that different environmental components—such as density, agricultural type of cultures, and spatial location of nest boxes—seem to be constraining the extent of plasticity on laying date (Bourret et al., 2015; see also Figure 2). Our results underlined that caution should be taken when aiming to generalize on the determinants of plasticity at the population scale. We also emphasized the importance of using a multidimensional framework (i.e., using several environmental variables) to address the consequences of plasticity on population response to environmental changes (Bourret et al., 2015).

Finally, in our eastern chipmunk study system we showed that adjustment in stress hormones (cortisol) levels in females are modulated by reproductive state, being lower during gestation and lactation (Montiglio, Garant, Pelletier, & Réale, 2015). We also found that intra‐individual variability in cortisol was also greater in females with smaller litters, again suggesting an interplay between extent of plasticity in ecological trait and potential for selection to act on that trait: For instance, stress reactivity may increase energy expenses and reduce reproductive success.

## 4 INTEGRATION AND FUTURE PERSPECTIVES

Over the next years, we will build upon our previous findings and recent theoretical and empirical advances to pursue three main interconnected research themes regarding the process affecting evolutionary changes in wild populations. In details, we will:

1. quantify strength of covariance between genetic variance and selection and its link to environmental variation;
2. describe plasticity evolution over time and space as the strength of selection acting on it; and
3. assess the importance of indirect genetic effects on fitness of individuals and on evolutionary potential of populations.

### 4.1 Covariance between genetic variance and selection

**Figure 3:**
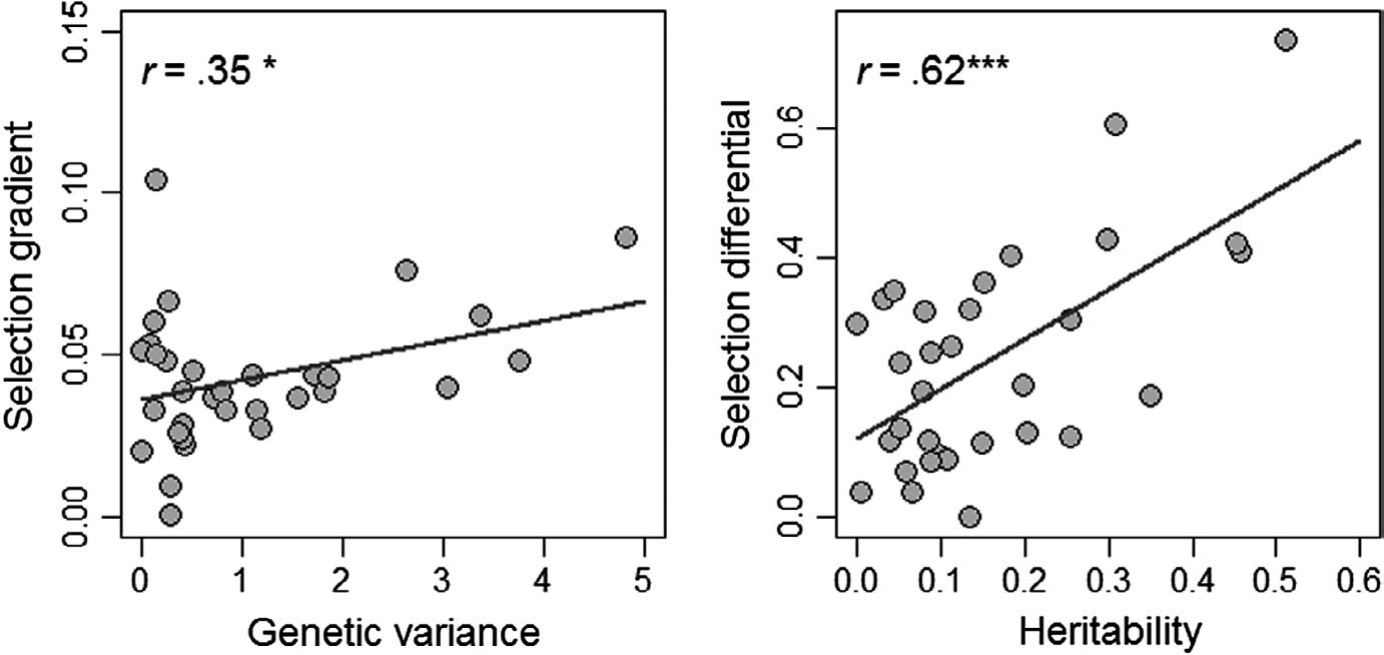
Correlations between (a) additive genetic variance and selection gradient and (b) heritability and selection differential for body mass of nestlings in Tree swallows. Each point represents estimates obtained for each year from 2007 to 2014 and at each age (either 2, 6, 12 or 16 days old). * *p* < .05, ***p < .001. See Bourret et al. (2017) for general methods

An underappreciated phenomenon concerning evolutionary potential of population is the possible effect of an environmentally driven correlation—positive or negative—between selection and heritable genetic variation acting on traits that could greatly affect their evolutionary response and adaptation rate. As suggested by Wood and Brodie (2016), in cases of positive correlation among parameters, it could either increase expected response to selection in the environments that generate strong selection and are characterized by abundant genetic variation or it could reduce it in the environments that produce weak selection and exhibit little genetic variation. In cases of negative correlation among parameters, the expected evolutionary response is always small but the constraint is different depending on if either the environments generating strong selection display little genetic variance (genetic variance is the limiting factor), or the environments that show weak selection exhibit abundant genetic variation (selection is limiting) (see Wood & Brodie, 2016 for details). Preliminary analyses in our study system suggest the presence of a positive covariance among these parameters (Figure 3). A recent study by Ramakers, Culina, Visser, and Gienapp (2018) used a meta‐analysis approach combining data from 16 long‐term population datasets providing 50 traits to assess the presence of such process in wild populations. They reported weak evidences for the presence of such covariation with little consequences in expected selection responses. The main problem however with their approach is that only two studies provided information about the environment itself, such that the authors had to use an indirect measure of environmental change in their analyses (i.e., mean trait‐value variation). There is thus a need for study with detailed environmental quantifications to reassess this pattern in the wild, which is what we aim to do over the next years. Another problem is that both genetic variance and selection are often not estimated annually on the same scale preventing the capture of true inter‐annual differences. To do so, one requires a large dataset in each year, such as the one we possess for Tree swallows nestlings (>500 in each year).

### 4.2 Plasticity evolution

Despite of its importance in the adaptive potential of a population, we still know little about heritability of plasticity and about how selection acts on plasticity (Merilä & Hendry, 2014). We will thus first document the variation in heritability and selection acting on plasticity across time and space. Genetic covariance among trait plasticity and the trait itself will also be documented to complement our analyses of the constraints or facilitation resulting from the genetic architecture of the traits. Also, as emphasized recently by Fox, Donelson, Schunter, Ravasi, and Gaitán‐Espitia (2019), there is a need to measure plasticity across both space and time. To do so, we will address to which extent within and transgenerational plasticity are linked, as they may depend on drastically different environmental conditions and “cues” (Miner, Sultan, Morgan, Padilla, & Relyea, 2005) and may be maladaptive under certain circumstances (Ghalambor, McKay, Carroll, & Reznick, 2007; King & Hadfield, 2019). Considering both time and space simultaneously is crucial as, for instance, recent evidences suggest that strength of between‐generation autocorrelation in the environment may generate maladaptive plastic response at one scale or the other (see King & Hadfield, 2019). Additionally, plastic responses to novel environmental conditions seem to mainly occur along trait dimensions possessing substantial amount of additive genetic variance (Noble, Radersma, & Uller, 2019). This makes it challenging to decipher if evolutionary responses are occurring because of plasticity or because of constraint imposed by genetic variance itself (Lind, Yarlett, Reger, Carter, & Beckerman, 2015; Schluter, 1996). Studies are thus needed to report on the main direction of plasticity though time and space and its relationships with amount of genetic variance reported for key traits along the same spatiotemporal dimensions. Furthermore, there is a requirement to understand more about the nature of selection on plasticity (Fox et al., 2019), and thus, we also need to measure what type and strength of selection act on plasticity. Recent evidences suggest that selection is mainly directional and weak (reviewed in Arnold, Nicotra, & Kruuk, 2019), but very few studies (based on only four species) have provided coefficients of selection for plasticity and thus more tests are needed before we can conclude on the importance of this process. To do so will require applying state‐of‐the‐art statistical analyses (see Arnold et al., 2019; Ramakers, Gienapp, & Visser, 2019) coupled with long‐term pedigree obtained from wild populations.

### 4.3 Indirect genetic effects

Social interactions are often an important part of an individual environment and can affect evolution and adaptation of populations through indirect genetic effects IGEs (where the phenotype of an individual is affected by the genotypes of others, which include maternal effects; McAdam, Garant, & Wilson, 2014; McGlothlin, Moore, Wolf, & Brodie, 2010; Moore, Brodie, & Wolf, 1997). In fact, IGEs can be expected in almost any system where conspecifics interact with each other (McAdam et al., 2014). For example, considering IGEs has changed interpretation about the causes of variation observed in several animal species and for several traits (reviewed in Bailey, Marie‐Orleach & Moore, 2018). There is currently a great deal of interest in research that explore the consequences of IGEs on phenotypes (including behavior, morphology, and life‐history traits—see e.g., Fisher et al., 2019) and their consequences on evolution. Considering IGEs help understand how evolution of traits may occur without adaptation, through changing fitness (Fisher & McAdam, 2019). For instance, if IGEs are negatively correlated with direct genetic effects (DGEs), then the population response to selection can be reduced, removed or even reversed, such as when traits are themselves dependent on the outcome of competition for limited resources (e.g., when food and/or density vary; Wilson, 2014). Yet, still very few studies conducted in the wild have considered interactions at the population level to assess both DGEs and IGEs and the extent of the correlation between them in contrasted environmental contexts (but see Fisher et al., 2019). We are currently assessing these processes in chipmunks and tree swallows.

## 5 MANAGEMENT IMPLICATIONS/RECOMMENDATIONS AND CONCLUSION

It is critical to tackle questions about the possible role of evolution in the current contexts of global warming and biodiversity crisis. Previous authors, such as Smith et al. (2014), have suggested that conservation practices in the Anthropocene require to integrate the concept of prescriptive evolution—that is the consideration of how evolutionary processes could be used to promote wise population management. For instance, Smith et al. (2014) suggested that prescriptive evolution could consist of management actions that would result in a better match between phenotypes of threatened species and their environment. Similarly, Carroll et al. (2014) suggested that management and conservation strategies could achieve great progress by slowing unwanted evolution of, for instance, pest species and/or by reducing phenotype–environment mismatch of species at risks from human‐induced changes. To achieve this will require documenting parameters such as amount of genetic variance for, and selection acting on, key traits as well of phenotypic plasticity of these traits. Studies combining multi‐species and multi‐disciplinary approach will be needed, and, in particular, studies using long‐term monitoring of marked individuals will continue to provide valuable data for biodiversity managers. Since we are expecting to maintain and expand such approaches in the next years, we hope our research will be helping getting a clear picture of the mechanisms behind responses to environmental changes, which is vital for the long‐term success of management and conservation actions.

## CONFLICT OF INTEREST

None declared.

## Data Availability

Data for this study were already deposited at Dryad: https://doi.org/10.5061/dryad.8vs6h and https://doi.org/10.5061/dryad.87jb3.
